# EpCAM homo-oligomerization is not the basis for its role in cell-cell adhesion

**DOI:** 10.1038/s41598-018-31482-7

**Published:** 2018-09-05

**Authors:** Aljaž Gaber, Seung Joong Kim, Robyn M. Kaake, Mojca Benčina, Nevan Krogan, Andrej Šali, Miha Pavšič, Brigita Lenarčič

**Affiliations:** 1 Department of Chemistry and Biochemistry, Faculty of Chemistry and Chemical Technology, University of Ljubljana, Večna pot 113, Ljubljana, SI 1000 Slovenia; 2 Department of Bioengineering and Therapeutic Sciences, Department of Pharmaceutical Chemistry, California Institute for Quantitative Biosciences, University of California, San Francisco, 1700 4th Street, Suite 503B, San Francisco, CA 94158 USA; 3 J. David Gladstone Institutes, San Francisco, CA 94158 USA; 4 Department of Synthetic Biology and Immunology, National Institute of Chemistry, Hajdrihova 19, Ljubljana, SI 1000 Slovenia; 5 Quantitative Biosciences Institute, QBI, Department of Cellular and Molecular Pharmacology, University of California, San Francisco, San Francisco, CA 94158 USA; 6 Department of Biochemistry, Molecular and Structural Biology, Institute Jožef Stefan, Jamova 39, Ljubljana, SI 1000 Slovenia

## Abstract

Cell-surface tumor marker EpCAM plays a key role in proliferation, differentiation and adhesion processes in stem and epithelial cells. It is established as a cell-cell adhesion molecule, forming intercellular interactions through homophilic association. However, the mechanism by which such interactions arise has not yet been fully elucidated. Here, we first show that EpCAM monomers do not associate into oligomers that would resemble an inter-cellular homo-oligomer, capable of mediating cell-cell adhesion, by using SAXS, XL-MS and bead aggregation assays. Second, we also show that EpCAM forms stable dimers on the surface of a cell with pre-formed cell-cell contacts using FLIM-FRET; however, no inter-cellular homo-oligomers were detectable. Thus, our study provides clear evidence that EpCAM indeed does not function as a homophilic cell adhesion molecule and therefore calls for a significant revision of its role in both normal and cancerous tissues. In the light of this, we strongly support the previously suggested name Epithelial Cell Activating Molecule instead of the Epithelial Cell Adhesion Molecule.

## Introduction

Epithelial Cell Adhesion Molecule (EpCAM) is a cell-surface type I transmembrane glycoprotein, which was initially identified as a colorectal carcinoma antigen^1,2^. Due to its elevated expression levels on carcinoma cells^3^ it is widely accepted as a target for drug delivery as well as a molecular marker in tumor diagnostics and prognostics (reviewed in ref.^4^). Here, high EpCAM expression is often associated with poor prognosis^5–11^ and linked to cancer proliferation, migration and metastasis^12^. Besides this, EpCAM expression is also high on undifferentiated human embryonic stem cells^13–15^. The molecular mechanisms on which EpCAM’s function in normal and cancerous tissue is based are still not explained completely. However, two major roles have been described – cell proliferation-enhancing signaling, which has been extensively studied in recent years^16–18^, and cell-cell adhesion, that hasn’t received abundant attention since its first description more than two decades ago^19^ and which we are addressing here in detail.

**Figure 1:**
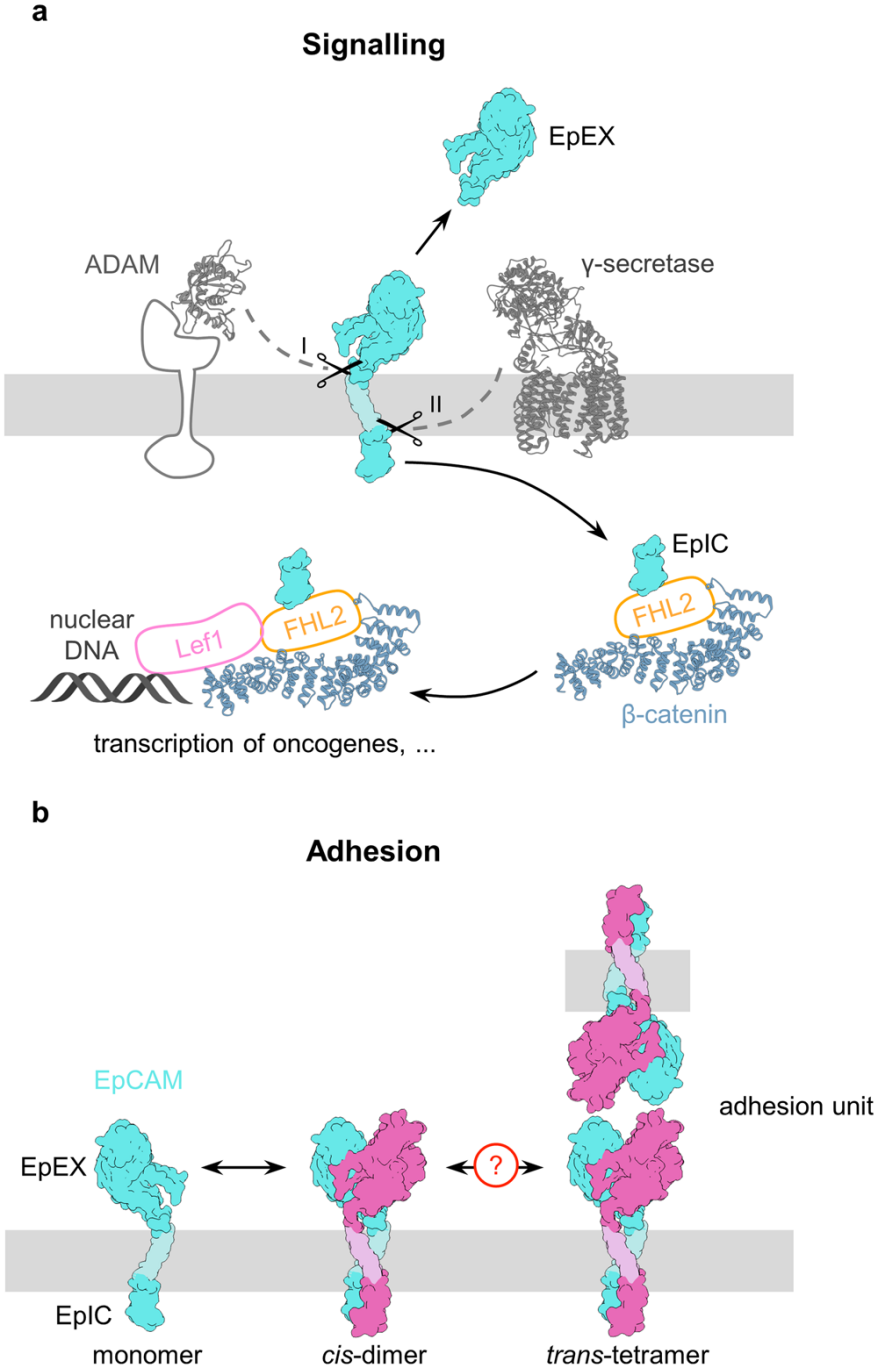
EpCAM’s function in cell-cell adhesion and signalling. (**a**) EpCAM mediated adhesion. EpCAM is depicted as a shape outline. Subunits in *cis*-dimers are colored cyan and magenta, transmembrane regions are depicted in paler colors. Cell membrane is shown in grey. (**b**) EpCAM signalling via RIP. EpCAM is depicted as a shape outline. Structures of ADAM’s catalytic domain, γ-secretase, β-catenin are presented as ribbons (PDB: 1bkc, 5a63 and 2z6h, respectively). The portion ADAM beside the catalytic domain, FHL2 and Lef1, whose 3D structures are not known are depicted as shapes with sizes corresponding to their mass.

Signaling via EpCAM involves regulated intermembrane proteolysis (RIP) (Fig. 1a), resulting in shedding of EpCAM’s extracellular domain (EpEX). Additional cleavages within the transmembrane region lead to release of the intracellular domain (EpIC) that is recruited into formation of EpIC-FHL2-Lef1-β-catenin complex that eventually binds to promoter region in the nucleus^16,20^. Through the interaction with Lef1, the EpIC-containing nuclear complex regulates the expression of proliferating factors such as cyclin D1 and *c-myc*^16,21^. RIP, the initial event in signaling, is believed to be triggered by *trans*-oligomerization of EpCAM’s extracellular domain, *i.e*. by binding of free EpEX to cell-bound EpCAM or via inter-cellular interaction between EpCAM molecules on adjacent cells^22^, as opposed to *cis*-oligomerization involving EpCAM molecules on the same cell. Alternatively, yet unknown ligand(s) of EpCAM may be instrumental in initiating RIP.

Oligomerization is also the principal feature of adhesion complexes formed during the establishment of proposed cell-cell contacts *via* EpCAM (Fig. 1b). Two models of inter-cellular oligomerization were proposed: formation of *trans*-octamers from *cis-*tetramers^23^ and, much later, formation of *trans*-tetramers from *cis*-dimers on adjacent cells^24^. The latter model is supported by the solved crystal structure of EpEX dimer^25^ in which the orientation of the two subunits is consistent with *cis*-orientation, and proposes a D2 symmetry of the *trans*-tetramer.

Initial discoveries describing EpCAM as a novel calcium independent homophilic cell adhesion molecule, including translocation to contact areas upon formation of cell-cell contacts^26^, disruption of the latter by EpCAM targeting antibodies^26^, and the ability to induce cell aggregation in transfected mouse fibroblast L929 cells^19^, were soon followed by indications that EpCAM’s role in cell-cell adhesion is much more complex. For example, EpCAM has a negative effect on the strength of classical E-Cadherin mediated cell-cell adhesion^27,28^ and directly interacts with claudin-7, thus regulating the formation of functional tight junctions^29,30^. Even more, despite notable evidence of EpCAM’s ability to directly mediate cell-cell adhesion, there have been some contradicting reports. First, a year after initial description of EpCAM as a novel CAM Fornaro *et al*. failed to reproduce EpCAM-induced cell aggregation^31^. Most recently, Tsaktanis *et al*. showed neither EpCAM cleavage nor EpCAM knockdown have any effect on cell-cell adhesion in a carcinoma cell line^20^. Furthermore, in our previous work with soluble EpEX^25^ we also failed to observe any evidence of higher-order oligomerization (*i.e*., more than dimerization). Combined, these findings encouraged us to reinvestigate the mechanism of EpCAM’s role as a cell-cell adhesion molecule (CAM) on a molecular level with an aim to provide a detailed explanation of the observed inconsistencies regarding the protein’s function.

Here we present structural analysis of EpCAM oligomerization and its ability to form higher-order oligomers, which would be consistent with the formation of homophilic cell-cell adhesion units. To gain insight into proteins behaviour both *in vitro* and *in vivo* we employed small angle X-ray scattering (SAXS), chemical cross-linking coupled with mass spectroscopy (XL-MS), bead aggregation assay (BAA), and fluorescence-lifetime imaging based Förster resonance energy transfer (FLIM-FRET). Our data clearly demonstrate that while both EpCAM and EpEX form *cis*-dimers *in vitro* and *in vivo*, no notable higher-order oligomerization takes place. Even more, EpCAM molecules from adjacent cells do not form inter-cellular higher-order homo-oligomers, making EpCAM’s function as a homophilic CAM highly implausible.

## Results

### EpEX cis-dimers do not form higher-order oligomers in solution

First we utilized SAXS on highly concentrated EpEX samples to see if high protein concentration leads to the formation of (significant) amount of higher-order oligomers which could be below the detection limit at low concentration, since the dissociation constant (*K*_d_) of the proposed *trans*-tetramer is much higher than the *K*_d_ of the *cis*-dimer (estimated values of 10 nM and 10 μM, respectively^24^). Here, formation of tetramers would manifest itself most prominently as increase in radius of gyration (*R*_*g*_), a measure of the average particle size. To reduce ambiguities in data interpretation and modeling, we utilized mutant non-glycosylated EpEX (ngEpEX) to avoid potential differences in glycosylation pattern in *wt* EpEX. Similarly, due to homogeneity issues we didn’t use full length EpCAM (EpFL) embedded in detergent micelles. Still, our results should be applicable to *wt* EpCAM since glycosylation reportedly does not interferes with EpCAM *trans*-oligomerization^23^ which is mediated exclusively via EpEX^23,25^.

**Figure 2:**
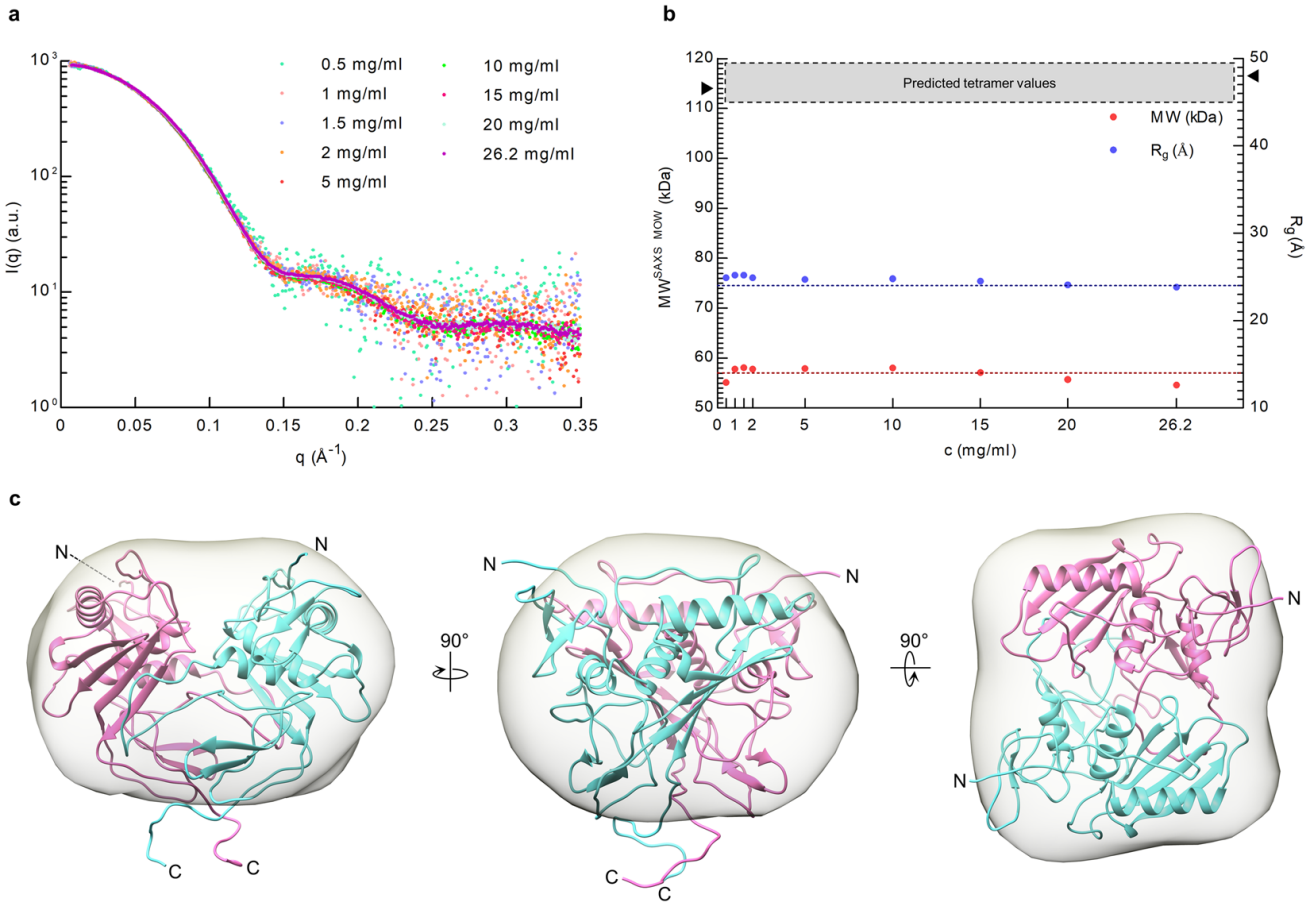
SAXS analysis shows no evidence of oligomerization. (**a**) Scaled SAXS profiles of ngEpEX at different concentrations in the range from 0.5 mg/ml (17.5 µM) to 26.2 mg/ml (919.4 µM) presented and overlaid in the same plot. (**b**) MW and *R*_*g*_ values calculated from SAXS profiles. Dotted lines represent values calculated for dimer structure 57 kDa and 24 Å. Predicted area of tetramer values are depicted with a grey rectangle and the hypothetical tetramer values at 114 kDa and 54 Å are marked with black triangles. (**c**) Three orientations of EpEX dimer structure docked in the a*b initio* shape (grey envelope) reconstructed from the merged SAXS profile. Subunits in the dimer are depicted as cyan and magenta ribbons.

SAXS data were collected at several protein concentrations ranging from 0.5 to 26.2 mg/ml (corresponding to 17.5–919.4 µM). At the highest sample concentration, approx. 93% of ngEpEX would be in tetrameric form considering a *K*_d_ of 10 μM and a simple dimer ↔ tetramer equilibrium relation, *i.e*. an amount that could most certainly be detected as changes in *R*_*g*_ *and D*_*max*_ (maximum particle size) values by SAXS analysis^32^. Surprisingly, we observed no significant concentration dependent changes, as the scaled SAXS profiles at different sample concentration nearly coincided (Fig. 2a). Furthermore, SAXS profile-derived MW and *R*_*g*_ values, both indirectly describing average particle size, correspond well to the values calculated from the slightly modified dimer crystal structure^25^ (PDB: 4MZV; C-terminal stretch of residues 259–265 was modelled as flexible). This indicates that the dimer is the highly predominant, if not the only oligomeric state of EpEX in the analyzed concentration range (Fig. 2b; Supplementary Table 1). This observation is further supported by the good size and shape agreement of the dimer X-ray structure with the *ab initio* shape reconstructed from the experimental SAXS profile (Fig. 2c).

To ensure radiation-preventing additives had no effect on the observed results, we collected SAXS profiles using two additional buffers (one buffer without glycerol, and another buffer containing 1% (w/v) sucrose and 5 mM Na/K Nitrate). While we did observe minor radiation damage in samples without any additives, SAXS profiles were highly consistent and comparable regardless of the buffer composition (Supplementary Table 1b,c).

Consistently, Multi-Fast open-source X-ray scattering (Multi-FoXS) analysis^33,34^ suggests that the population of the proposed *trans*-tetramer form is simply negligible, in all SAXS concentrations between 0.5 to 26.2 mg/ml. We first searched multi-state models from a combined ensemble of 10,000 dimer models (generated using Modeller^35^ from the crystal structure of PDB 4MZV by accounting for flexible C-terminus) and 44,098 tetramer models (generated using Patchdock^36,37^ to obtain all possible configurations of two dimer units forming a tetramer). Next, we compared the calculated SAXS profiles of the multi-state models with the experimental SAXS data by using the χ^2^ score value. The best fit of the resulting multi-state models did not significantly improve over the single-state dimer structures (Supplementary Fig. 1). The single-state dimer structures are highly consistent with the experimental SAXS data up to 15 mg/ml (χ^2^ values of 1.39–1.97, Supplementary Fig. 1). Although minor inter-molecular repulsion was observed at higher protein concentrations (>15 mg/ml), it does not change any conclusion from the Multi-FoXS analysis. All resulting multi-state models that did improve the fit were solely comprised of dimer structure models with minor conformational rearrangements. In contrast, the tetramer structure models were completely excluded from the 100 best scoring multi-state models at all concentrations.

Collecting additional data at even higher protein concentrations than 26.2 mg/ml was not feasible since we observed the inter-molecular repulsion already at ~15 mg/ml. The repulsion is strongly indicated by smaller protein MW and *R*_*g*_ values (Supplementary Table 1a), supporting that at the high concentration ngEpEX dimers tend to repel each other rather than attract to form oligomers. All these combined results thus clearly suggest that EpEX dimers do not interact to form higher-order oligomers in solution.

### Assignation of chemical cross-links as input data for structural modeling

Appearance of bands corresponding to the molecular mass of a dimer and tetramer in chemically cross-linked EpCAM samples is considered one of the fundamental proofs of EpCAM’s adhesion-mediating oligomerization^23,24,38^. To see if such data can be used as distance restraints in structural modeling of the proposed tetramer we used chemical cross-linking coupled with mass spectroscopy (XL-MS) to identify the cross-links within electrophoretically-separated bands corresponding to the different oligomeric forms of EpCAM. This approach would at the same time enable us to capture transient oligomers that could be too short lived to be detected by SAXS. For these experiments we used both ngEpEX as well as ngEpFL thus addressing the observation that transmembrane and intracellular part are necessary for formation of higher-order oligomers^24^. *Wt* glycosylated EpCAM was not used to avoid issues with heterogenic glycosylation during cross-link assignation by MS.

**Figure 3:**
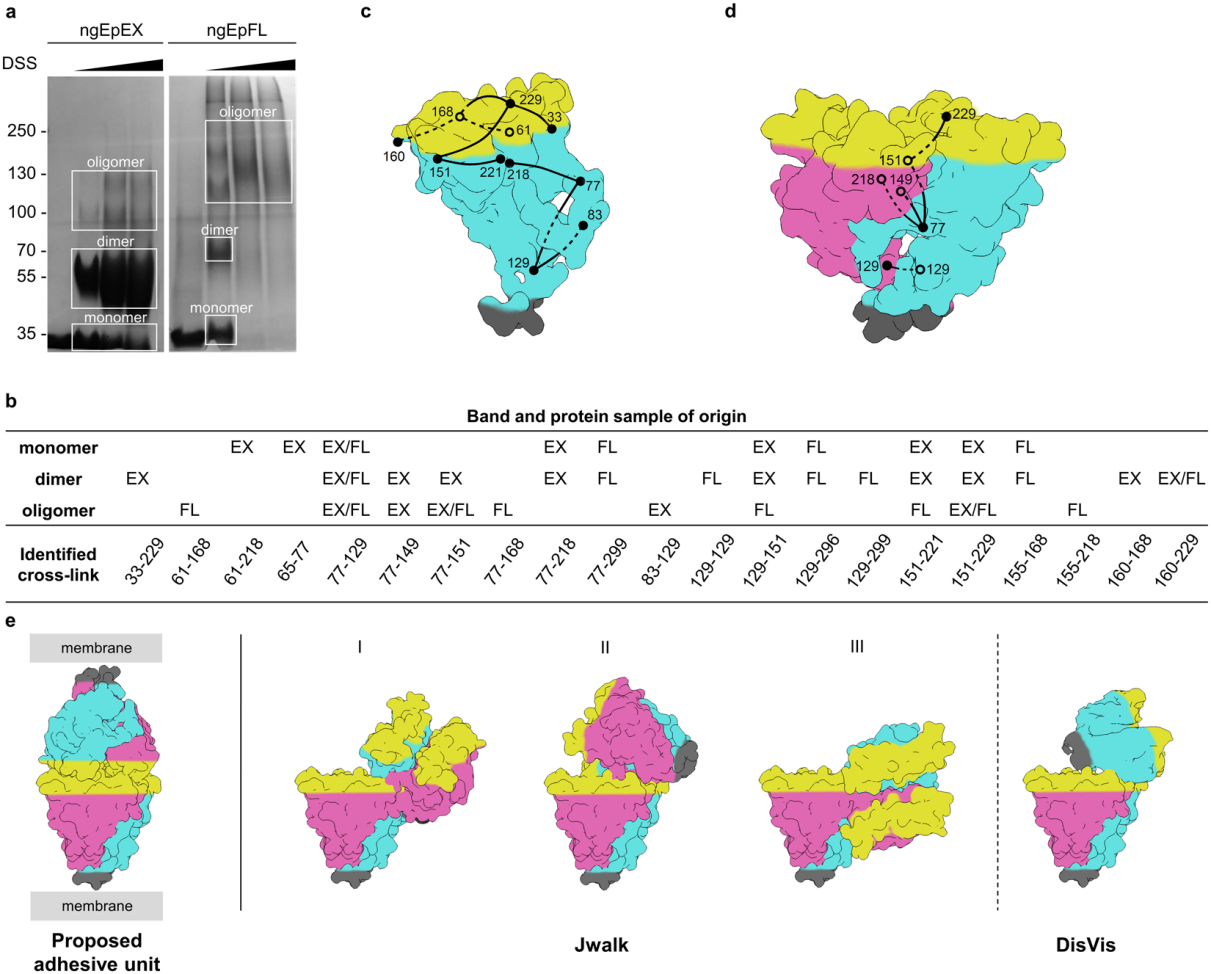
XL-MS analysis of EpCAM oligomerization. (**a**) SDS-PAGE analysis of cross-linking experiment. Black triangle at the top indicates increasing molar ratio of crosslinker (DSS) *vs* protein. White rectangles denote excised areas, which were analysed with MS. Lanes were cropped and greyscaled for clarity. Full-length gel is presented in Supplementary Fig. 3. (**b**) Cross-links identified in MS analysis. Columns represent corresponding areas of origin, EX and FL stand for identification in ngEpEX or ngEpFL XL experiment, respectively. (**c**,**d**) Shortest SASD for each matched cross-link in monomer model and dimer structure. In dimer structure, only one of each ambiguous inter-dimer cross-links is represented. Subunits are depicted as shape outlines. Subunits in *cis*-dimers are colored cyan and magenta, with membrane proximal and membrane distal parts depicted in grey and yellow, respectively. (**e**) Modelling of tetramers based on cross-links, non-accessible in dimer. Proposed *trans*-tetrameric adhesive unit model was taken from literature (Pavšič, 2014). Jwalk results are presented as three clusters, generated from 25 best scoring random tetramer models, clustered at 10 Å. DisVis results represent average ligand occupancy at default cut-off, as outputted by the web server.

In agreement with previously published findings^23,24^, four distinct bands were observed after SDS-PAGE analysis of cross-linked samples. Their masses correspond to mono-, di-, tri- and tetrameric ngEpEX or ngEpFL (Fig. 3a). Cross-linking was more efficient in the case of ngEpFL, resulting in higher percentage of higher-oligomeric forms compared to ngEpEX where the most intense band corresponds to a dimer (regardless of cross-linker concentration). Separate bands were excised, in gel digested, and resulting peptides separated and measured by LC-MS/MS. Using the cross-linked peptide analysis feature of Protein Prospector^39^ we identified 21 unique cross-links (Fig. 3b). For structural modeling, we considered only the 18 cross-links which had both endpoint residues in the ectodomain (cross-links 77–299, 129–296 and 129–299 were excluded since they involved the intracellular region of ngEpFL). Two cross-links were obtained exclusively from bands corresponding to a monomer, four to a dimer, and four to a higher-order oligomer, while the rest (n = 8) were identified from bands corresponding to more than one oligomeric state. Surprisingly, we did not observe any cross-links exclusively within the IC-tail, despite the abundance of lysines (5 out of 23 residues).

Unambiguous assignation of cross-links as intra- or inter-subunit within the various oligomeric states is not always possible, regardless of the band of origin on SDS-PAGE. To avoid false assignation, we assumed that all obtained cross-links can belong to any oligomeric form. The only exception were cross-links obtained exclusively from monomeric bands; these were postulated to be intra-subunit since they would otherwise cause significant shift in electrophoretic mobility (cross-links 61–218 and 65–77). All other cross-links were assigned to the oligomeric state in which they had the highest “Matched and Non-accessible cross-link score” (MNXL score) calculated from their Solvent Accessible Surface Distances (SASDs)^40^. MNXL score distinguishes between experimentally observed cross-links, which have SASD lower than empirically determined threshold for DSS cross-linker (33 Å) – “matched cross-links” and all those who have larger SASDs or aren’t present on the solvent accessible surface of the protein/protein complex – “non-accessible cross-links”. Scoring based on SASDs was used to avoid false identification of cross-links as intra-subunit as is often the case with the more commonly used Euclidean distances.

First, we calculated SASD for each cross-link and assigned them according to the published parameters using an adapted version of Jwalk^40^ (see Materials and Methods for details). Distances were calculated for 17 cross-links in the subunit structure (total dataset excluding 129–129, which can’t arise from an intra-subunit cross-link), and 16 cross-links in the dimer structure (total dataset excluding 61–218 and 65–77, which appeared only in bands corresponding to a monomer). For monomer, 9 cross-links could be matched while 8 had SASD larger than 33 Å (Fig. 3c; Supplementary Fig. 2). To account for monomer flexibility on a small scale (sidechains plus limited main chain conformational changes), equivalent SASDs derived from 1000 frames in 10 ns MD simulation (one frame per 10 ps) were calculated. Of the 8 non-accessible, two more (65–77 and 155–168) could be matched and two had distances just above the threshold of 33 Å (61–218 and 77–151) (Supplementary Fig. 2). In dimer structure, considering both intra- and inter-subunit distances, 12 of 16 cross-links were matched while 4 SASDs were longer than the threshold (Fig. 3d; Supplementary Fig. 2). They correspond to cross-links 77–168, 129–151, 155–168 and 155–218.

### Cross-links do not support a functionally relevant trans-tetramer model

After cross-link assignation four aforementioned cross-links remained which could not be matched neither to monomer nor to the *cis*-dimer structure. Therefore, we assumed that they stem from a tetrameric form of ngEpEX/ngEpFL. To see if they could indeed support a functionally relevant *trans*-tetramer model we performed an exhaustive explorative search of virtually all possible tetramer models with an aim to match those four remaining cross-links. At the same time, we attempted to see whether alternative structural models improve MNXL scores of the already matched cross-links.

The first step was the generation of a large set of virtually all possible random tetramer models (using a dimer crystal structure). This approach ensures that all possible dimer-dimer orientations are tested for their consistency with the experimental data in contrast to using spatial restraints from cross-links to guide the sampling from the beginning. Next, inter-dimer SASDs were calculated for the 44,098 random tetramer models (which were also used for multi-FoXS analysis above) and 12 D2 symmetric *trans*-tetramer models and compared them to corresponding intra- and inter-subunit SASDs in the dimer. None of tetramer models significantly improved the results for any cross-link already matched in the dimer, however some tetramer models can be used to match all four remaining non-accessible cross-links (Supplementary Fig. 2b). Importantly, random tetramer models outperform models with D2 symmetry in terms of satisfying the cross-link-derived distance restraints. Best tetramer model with D2 symmetry, i.e. model with the highest MNXL score, matched only two of the four previously non-accessible cross-links (Supplementary Fig. 2b).

To assess their functional significance the top-scoring models were compared to a proposed adhesion unit with respect to C-termini orientation corresponding to the membrane anchor point. For clarity, they were first clustered considering their symmetry with a 10 Å RMSD-cutoff. In none of the three resulting clusters the two dimers are oriented in a way that could even approximate a theoretically plausible *trans*-interaction between dimers from adjacent cells (Fig. 3e).

Similar results were obtained using DisVis^41,42^ which uses Euclidean rather than solvent accessible surface distances. DisVis also accounts for the possibility that some of the obtained cross-links are false positives. We preformed the analysis with the four cross-links that were non-accessible in the dimer. The results again indicate that the obtained tetramer models could not represent a functionally relevant trans-tetrameric adhesion unit (Fig. 3).

### Neither avidity nor native-like orientation can facilitate the formation of trans-tetramers

**Figure 4:**
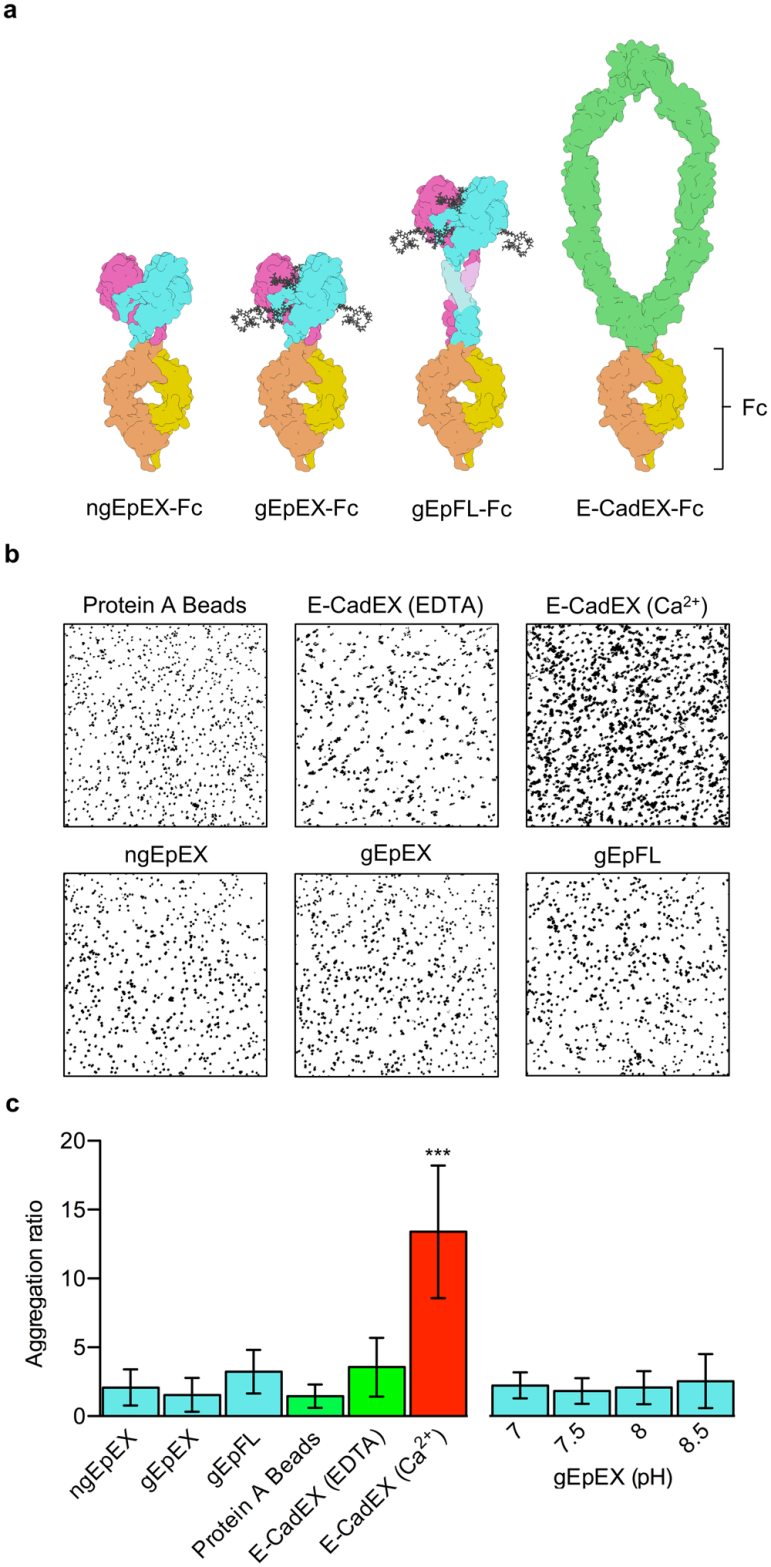
Bead Aggregation Assays. (**a**) Schematic representation of analysed proteins. Subunits in EpEX and EpFL are depicted as in Fig. 1. Glycosylation is depicted as grey sticks. E-CadEX dimer is green and Fc-dimer is orange and yellow. (**b**) Representative bead aggregation images after image analysis with ImageJ. Bead aggregation is seen as clustering of black spots. (**c**) Comparison of Aggregation ratios. Values represent mean values of 15 independent images with s.d., ***p < 0.001, one-way ANOVA test with Bonferroni post hoc analysis.

The bead aggregation assays (BAA) represents a simple and robust way to assess homo-oligomerization of proteins. Because of its advantages in terms of providing avidity as well as possibility to mimic natural orientation(s), this approach has been often used to characterize cell-adhesion molecules^43–46^. We have performed BAAs with various EpCAM variants all C-terminally fused to hinge-and-Fc regions of human IgG (Fig. 4a). The purpose of the fusion is dual: (1) by locking the two EpCAM subunits in close proximity via a di-sulfide bond in the FC regions it stabilizes the *cis*-dimer and thereby ensures that only higher-order oligomerization can facilitate the aggregation of beads, and (2) binding of the Fc region to the bead-immobilized Protein A orients EpCAM in a correct, native-like orientation, where extracellular domains point outwards of the surface. The EpCAM variants used were ngEpEX and gEpEX (thereby considering also the glycosylation, albeit non-native as produced in insect cells), and gEpFL; ngEpFL was not used since the Fc-fusion could not be obtained in sufficient amounts. As a positive control an Fc fusion of extracellular domain (EC1-EC5) of human E-Cadherin was used (E-CadEX-Fc) - such fusion has been previously reported to successfully induce bead aggregation^43,44^.

Results indicate that none of the used forms of EpCAM can induce any significant bead aggregation, while E-CadEx-Fc induced bead aggregation in presence of calcium ions, which was blocked upon treatment with EDTA (Fig. 4b). Bead aggregation ratios of EpEX fusions are not significantly different than those of Protein A beads only (Fig. 4c), suggesting EpCAM dimers attached to separate beads do not interact through their extracellular domains in a homophilic manner. Slightly increased aggregation ratio was observed in the case of gEpFL-Fc, however not statistically significant and only comparable to the ratio of E-CadEX-Fc in the presence of EDTA that is physiologically irrelevant. Considering the protein aggregation during gEpFL-Fc purification, this bead aggregation is probably mediated through TM-region embedded in micelle and is not the consequence of interactions between extracellular domains.

Aggregation of gEpEX was further analyzed at various pH values and without calcium ions to ensure all relevant physiological conditions were considered, however none of those conditions resulted in any significant bead aggregation (Fig. 4c).

Taken together, bead aggregation assay is in line with our previous observations that EpCAM *cis*-dimers do not interact in a manner that could result in formation of cell-cell contacts (i.e. *trans*-dimerization of *cis*-dimers on neighboring cells). Furthermore, even avidity, inherent by design in BAA, does not contribute to formation of such inter-bead higher-order oligomers. This indicates that neither correct native-like orientation nor high local concentration of EpCAM molecules can facilitate the formation of *trans*-tetramers.

### EpCAM forms cis-dimers but not trans-oligomers *in vivo*

To analyze homophilic interactions between EpCAM molecules in their native setting, i.e. cellular environment, including native-like glycosylation, we employed FRET to probe the distances between various fluorescently-labeled EpCAM variants. In general, donor and acceptor fluorophores attached to the proteins under study have to be no more than 10 nm apart for FRET to occur. To date, several FRET-based methods have been developed to measure direct protein-protein interactions in living or fixed cells^47^ and in a properly designed experiment, the presence of FRET is considered a direct indication of protein-protein interaction. For our analysis we used Fluorescence Lifetime Imaging Microscopy (FLIM-FRET), which is a very robust method because variations in excitation intensity, inner filtering, moderate donor photobleaching and detector sensitivity do not influence fluorescence lifetime^48^. In FLIM-FRET, existence of FRET can be inferred from a drop of fluorescence lifetimes (τ), as the presence of acceptor fluorophore offers an additional deactivation pathway from the donor-excited state^48^.

**Figure 5:**
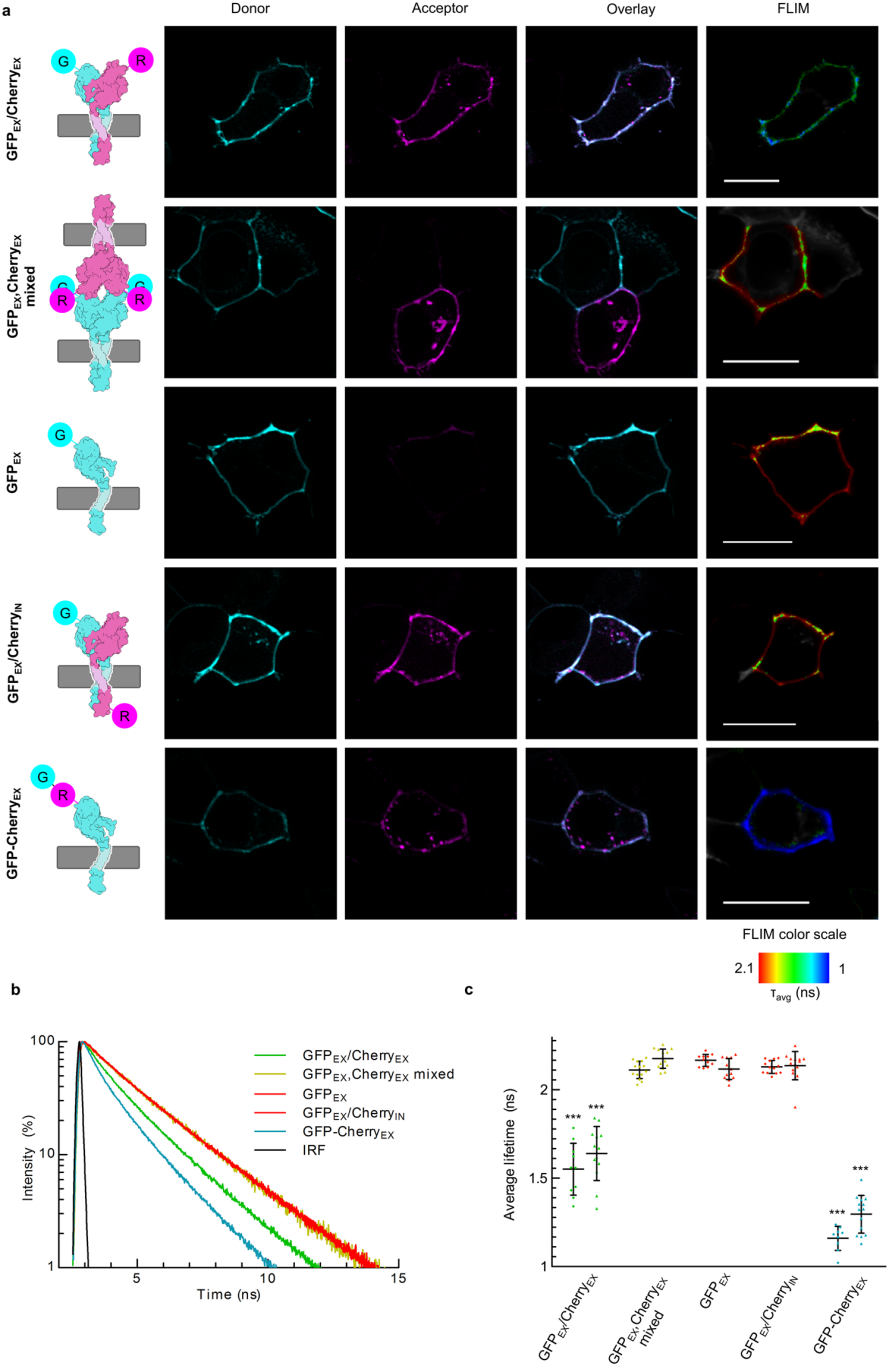
FLIM-FRET (HEK 293 T). (**a**) Analysed combinations of fluorescently tagged EpCAM proteins. sfGFP (G) fluorescence is colored cyan and mCherry (R) fluorescence is colored magenta. The same color scheme applies to schematic representations on the left. White line represents 20 µm. FLIM color scale is the same in all presented FLIM measurements. (**b**) Representative fluorescence lifetime decays for each analysed combination. (**c**) Mean lifetimes with s.d. of all analysed combinations. For each combination, results obtained in HEK 293 T cells are presented on the left and results obtained in HCT8^EpCAM−^ on the right, ***p < 0.001, one-way ANOVA test with Bonferroni post hoc analysis, compared to mean values of negative controls. Color scheme is the same as in figure b.

We observed lifetimes of different combinations of N- or C-terminal EpCAM fusions with an EGFP variant sfGFP or/and mCherry. EGFP variants and mCherry are well characterized FRET pairs, suitable for FLIM with r_0_ = 5.4 nm^49^. The distance between N-terminals in *cis*-dimer (~7 nm) and the maximal distance between N-terminals in a *trans*-tetramer (~5 nm, assuming a D2 symmetric *trans*-tetramer with maximal, 90° angle at y-axis) fall within the measurable FRET range for this FRET pair. Donor lifetimes were measured in cells transfected with either N-terminal donor only (GFP_EX_) to measure τ of donor fluorophore when FRET is not possible, N-terminal donor covalently linked to N-terminal acceptor on the same protein (GFP-Cherry_EX_), to assess the maximal drop of τ due to FRET, and N-terminal donor and C-terminal acceptor transfected in the same cell (GFP_EX_/Cherry_IN_) as a negative control for distances exceeding FRET range. Next, we measured donor life times in N-terminal donor and N-terminal acceptor transfected in the same cell (GFP_EX_/Cherry_EX_) to observe intra-cellular *cis*-oligomerization, and of mixed cells transfected with either donor only or acceptor only, to form donor-acceptor pairs at the areas of cell-cell contacts (GFP_EX_, Cherry_EX_ mixed), which would result in FRET due to inter-cellular *trans*-oligomerization (Fig. 5a). Two different EpCAM negative cells lines were used to observe potential changes in different cell types: human epithelial cell line HEK 293 T (Fig. 5a,b) and CRISPR/Cas EpCAM-knockout colorectal adenocarcinoma cell line HCT8 #L13^20^ (Supplementary Fig. 4).

In our case, GFP_EX_ exhibited biexponential fluorescence decay with average lifetime of 2.14 ± 0.05 ns (Fig. 5c). Significant drop in the average lifetime (τ_avg_ = 1.2 ± 0.1 ns) was observed in case of covalently linked donor and acceptor (Fig. 5c). As expected, no change in donor lifetime was observed in GFP_EX_/Cherry_IN_ (τ_avg_ = 2.13 ± 0.06 ns) (Fig. 5c), where FP are on the opposite site of the membrane, too far apart for FRET. On the other hand, GFP_EX_/Cherry_EX_ exhibited average lifetime of 1.6 ± 0.15 ns, indicating EpCAM does form cis-oligomers *in vivo* (Fig. 5c). To answer whether the observed difference in the lifetime is a result of dimerization or even higher-order oligomerization, further experiments would be required.

When cells expressing either sfGFP-EpFL or mCherry-EpFL were mixed (GFP_EX_, Cherry_EX_ mixed), no statistically significant change in lifetime was observed at the contact area (τ_avg_ = 2.14 ± 0.06 ns) (Fig. 5c). To ensure fixation was not the cause for the lack of observed inter-cellular FRET, this experiment was also performed in live HEK 293 T cells, yielding similar results (Supplementary Fig. 5a). Furthermore, lifetimes at cell-cell contact areas did not significantly differ from lifetimes of areas where only donor FP was present (Supplementary Fig. 5b). This provides evidence that although EpCAM may be localized at the areas of the cell-cell contact as reported previously, EpCAM molecules on the opposing cells are not sufficiently close nor involved in a direct interaction.

## Discussion

Initial analysis of the function of EpCAM determined a role in cell-cell adhesion^19,26^ through homophilic interactions^23,24^. These assigned functions led to its name as epithelial cell adhesion molecule. Ever since and despite seemingly contradicting observations regarding its role in formation of cell-cell contacts^20,25,31^. EpCAM’s ability to form adhesion units through homo-oligomerization of extracellular domains was well accepted. The results generated by our comprehensive approach using a variety of methods, constructs (full-length and truncated form of EpCAM, with/without fusion) and their variants (non-glycosylated, non-native glycosylated and native glycosylated) both under *in vitro* and *in vivo* conditions present strong evidence that such interactions are highly unlikely and that EpCAM thus cannot function as a homophilic CAM, at least not in a manner in which it was initially proposed^23,24^.

The SAXS analysis of EpEX oligomeric state in solution clearly demonstrates that the EpEX exists predominately in its dimeric form (Fig. 2). First, both shape and size as well as comparison of calculated SAXS profiles with experimental SAXS profiles (Fig. 2; Supplementary Fig. 1) confirm the initial assumption that the dimer observed in the crystal structure represents the actual structure of the dimer in solution^25^. Furthermore, this provides additional support for the assumption that some of the initial studies on EpCAM oligomerization, claiming that EpEX is a monomer up to a concentration of 6 mg/ml^24^, were premature. We can only speculate what contributed to such discrepancy in results; one possible reason is that the initial conclusion was based on sedimentation equilibrium analysis which lacks sufficient sensitivity as compared to SAXS. While there could potentially still be an equilibrium between monomeric and dimeric form even at low concentrations, EpEX clearly does not partake in formation of any higher-order oligomers consistent with its function as a homophilic CAM (Supplementary Fig. 1). Although such analyses of soluble ectodomains of adhesion proteins do not completely represent the conditions *in vivo*, the applicability of results to full-length proteins have been demonstrated in many other similar cases including Cadherins^50–52^, PECAM-1^53^, NCAM^54^, JAM-A.^55^, L1^56^, Nectin^57^, Desmocollins and Desmogleins^58^. This is not a surprise as large cell-cell interaction-mediating ectodomains of type I transmembrane adhesion proteins frequently represent the major portion of the protein. Function of the transmembrane region and the intracellular domain is, in this respect, mainly to anchor the protein to the membrane and the cytoskeleton, which was similarly claimed for EpCAM^59^. Furthermore, consideration of concentrations used in our SAXS analysis with regard to *K*_d_ values of inter-cellular interactions of other adhesion proteins confirms that the concentrations were high enough to enable formation of functionally-relevant higher-order oligomers. In the light of this we did not observe any sign of oligomerization at concentrations roughly an order of magnitude higher than the *K*_d_ for oligomerization of E-Cadherin ectodomain^50,51^, which is considered as one of the weaker cell-cell interactions.

The only method used to date to study both structure and stoichiometry of EpCAM oligomers was chemical cross-linking^23,24^. We have included it also in our repertoire, however we have supplemented it with MS-based cross-link identification. This enabled us to match the identified cross-links to subunit and dimer structure of EpCAM as well as to the models of the proposed tetramer. As already reported in the initial studies, we have also observed bands corresponding to molecular mas of di-, tri- and tetramer after cross-linking either EpEX or EpFL (Fig. 3a). Presence of bands corresponding to tri- and tetramer is not in agreement with SAXS experiments which do not indicate higher-than-dimer oligomeric forms in solution at the same protein concentration (1 mg/ml). This calls for careful investigation whether the apparent presence of higher-order oligomers is a consequence of cross-linking artefacts or a result of biologically relevant interactions. Our structural modelling based on spatial restrains obtained from XL-MS suggest the cross-linking artefacts since the best model explaining the experimental data is based on random tetramers without the ability to support formation of the postulated cell-cell contacts (Fig. 3e). The bands corresponding to trimers were separately used for structural modelling as they can be assumed to stem from partially cross-linked tetramers. However, due to limitations of modeling of homo-oligomeric proteins based on cross-link-derived distance restraints, namely the oligomeric state ambiguity of identified connections and the possibility of false positives, a definite disproval of *trans*-tetramer existence is not possible. *Trans*-tetramers could be present in our samples however their identification was hindered due to beforementioned restrictions or the fact that the *trans*-tetramer specific cross-links simply weren’t detected in MS. Still, our extensive analysis clearly demonstrates tetramer existence cannot be directly implied based solely on apparent molecular weight of cross-linked species, as it was done in previous reports^23,24^.

To sum up, relatively high concentrations of cross-linker needed to observe significant bands corresponding to tetramer of ngEpEX and the fact that all four cross-links not found in subunit and dimer can be better explained by random interactions between two dimers indicate that the appearance of the band corresponding to higher-order oligomers is more likely an artefact than a consequence of functionally relevant *trans*-tetramers.

Similarly, no aggregation was observed in BAA using either ngEpEX-Fc or gEpEX-Fc. While dimerization via di-sulfide bonds in Fc-regions ensures EpEX subunits are covalently linked in a dimer, it could also impair proper formation of the EpEX *cis*-dimer, which is a prerequisite for aggregation via the formation of a *trans-*tetramer. However, due to the inherent flexibility of hinge regions in IgGs^60^ and the stability of EpEX *cis*-dimer demonstrated in this and other research^25^, and matching distance between N-terminals in FC-region dimer and N-terminals in EpCAM transmembrane region models, we believe it is highly unlikely proper formation of the EpEX *cis*-dimer would be completely abolished. Furthermore, such potential structural hindrance would not affect proper dimerization of gEpFL in gEpFL-FC fusion, where EpCAM’s transmembrane region and EpIC are located between the EpEX and the hinge-and-FC region. Small, statistically insignificant, difference from negative control aggregation ratio could be detected using gEpFL-Fc (Fig. 4c,d). Since neither ngEpEX-Fc nor gEpEX-Fc induced any bead aggregation at all, this can’t be attributed to *trans*-interactions between ectodomains. The more likely explanation is that EpFL is prone to *cis-*aggregation. This hypothesis is in agreement with previously published results where the presence of bands corresponding to tetramers in *in vivo* cross-linking experiment was observed even when single, non-interacting cells were examined (in this case the authors concluded the adhesion unit is a *trans*-octamer, without any direct evidence of its existence)^23^.

To place the system under study into cellular context we employed FLIM-FRET analysis, which did show evidence of *cis*-oligomerization (Fig. 5, Supplementary Fig. 4, experiment labeled GFP_EX_/Cherry_EX_). However, the experimental design prohibited us from elucidating the stoichiometry of interactions. Based on other results presented in the present and in previous publications^25^, we speculate that the observed change in donor lifetimes was a consequence of *cis-*dimerization. On the other hand, no evidence of *trans*-oligomerization even though both the donor and the acceptor labeled EpCAM on the opposing cells were colocalized at the area of cell-cell contact (Fig. 5, Supplementary Fig. 4, experiment labeled Cherry_EX_ mixed). Combined with our *in vitro* experiments discussed above, this provides conclusive evidence that EpCAM is not able to form inter-cellular *trans* interactions, which are prerequisite for its function as a homophilic CAM.

If EpCAM indeed is a CAM, our results show that its function like one can’t be a directly linked to its homo-oligomerization as it has been widely accepted for more than 20 years. Even more, we believe that the most feasible description of EpCAM is that it is in fact not a cell-cell adhesion molecule at all. Our results in combination with those previously reported by Tsaktanis *et al*.^20^ support this hypothesis both *in vitro* and *in vivo*. Furthermore, EpCAM shares no structural similarity to any other superfamily of known adhesion molecules. Also, the role in cell-cell adhesion has never been described for its closest homologue Trop2^31^ with which it shares 67% amino acid sequence similarity. It should also be noted that the Fornaro *et al*. failed to reproduce the initial results of EpCAM’s direct involvement in cell-cell adhesion in transfected murine fibroblasts^31^. Similarly, no effect on cell segregation upon transfection was observed in thymic epithelial cells^61^. Additionally, EpCAM mediated adhesion has been described as very weak; therefore, zipper- or cluster-like extensive contacts would be expected to compensate for weak single inter-cellular interactions via avidity, however they have never been observed^23,62^.

Our results clearly demonstrate that EpCAM partakes in *cis-*oligomerization *in vivo*, however since no other interactions between ectodomains exist both *in vitro* and *in vivo* some aspects EpCAM biology should be revisited in the future. First, the role of alleged *trans*-interactions in inducing RIP should be addressed in detail. If homophilic interactions as believed to date^16^, aren’t the cause for its initialization, we speculate that an interaction with a yet unidentified ligand might be involved. Also, it has been already demonstrated that the dimeric form would structurally obstruct cleavage by ADAM and BACE1 during RIP^20^. Similarly, dimerization of the substrate has also been shown to hinder processing by γ-secretase in the case C-terminal fragment of the amyloid β protein-precursor (APP CTFβ)^63^. In the light of this the role of the EpCAM *cis*-oligomerization and its regulation, particularly with regard to its interaction with other proteins, still remains to be studied in detail.

Even without direct involvement in formation of cell-cell contacts, EpCAM remains an important mediator of cell-cell adhesion, since it is involved in negative regulation of both classical E-Cadherin mediated cell-cell adhesion^27,28^ and positive regulation of tight junction formation via a direct interaction with claudin-7^29,30^). These interactions combined with its ever more evident signaling function are still sufficient to explain its diverse role in epithelial morphogenesis^64^, organization^65^ and cancer^12^. Therefore, we believe that properly addressing the function of EpCAM, including refuting its property as a homophilic CAM, will help in elucidating its various functions. To avoid misconception, we also support that the molecule is renamed to Epithelial Cell Activating Molecule, as previously suggested^20,66^.

## Methods

### Protein cloning, expression and purification

ngEpEX and gEpEX were expressed in insect cell line *Spodoptera frugiperda* (Sf9, Thermo Scientific, USA) and purified as described before^25^.

EpFL forms were expressed in similar manner. 3 days post transfection with baculoviral stock, cells were collected by centrifugation for 10 min at 10,000 × g, resuspended in 20 mM HEPES, pH 7.5, 150 mM NaCl, and pelleted again. The pellet was then resuspended in 50 mM HEPES, pH 7.5, 150 mM NaCl, 20% (v/v) glycerol, 10 mM EDTA, 1 mM PMSF, and lysed using a Teflon homogenizer. Membrane fraction was collected by centrifugation for 1 h at 40,000 × g. To solubilize EpFL, 50 μl/10^6^ cells of extraction buffer containing 20 mM HEPES, pH 7.5, 150 mM NaCl, 20% (v/v) glycerol, 2% (v/v) Triton X-100 was added. The suspension was stirred for 1 h at 4 °C. Insoluble fraction was removed by centrifugation for 1 h at 40,000 × g. Soluble fraction was applied to cOmplete™ His-Tag Purification Column, which was previously equilibrated using 20 mM HEPES, pH 7.2, 300 mM NaCl, 15% (v/v) glycerol, 0.05% (v/v) Triton X-100 (reduced form). Elution was done using an imidazole gradient (final concentration of 500 mM). Fractions containing EpFL were dialyzed against the binding buffer overnight and then reapplied to the same column. After elution and dialysis using the same procedure as previously, samples were concentrated and purified using size-exclusion chromatography column (Superdex 200) equilibrated using 10 mM HEPES, pH 7.5, 250 mM NaCl, 0.05% (v/v) Triton X-100 (reduced form).

EpEX or EpFL fused to IgG1 heavy chain hinge and Fc region (both derived from ps521-hEpEX-Fc^20^, a kind gift from Olivier Gires) and with added C-terminal His_6_-tag were cloned into pFastBac1. Following transposition to generate recombinant bacmids in *E. coli* DH10MultiBac cells the recombinant bacmids were isolated and used to transfect *S. frugiperda* Sf9 cells. The process of baculovirus preparation and protein expression was the same as described before^25^. For isolation, only one immobilized metal affinity chromatography and subsequently one size exclusion chromatography step was performed. Buffers were the same as the corresponding buffers used for either EpEX or EpFL.

E-CadEX-Fc fusions were designed with fusion to human E-Cadherin (aa 1–709), cloned from hE-cadherin-pcDNA3^67^ (a kind gift from Barry Gumbiner, Addgene plasmid #45769), and IgG1 heavy chain hinge and Fc-region (residues 105–330). E-CadEX-Fc was cloned into pFastBacDual-Furin under polyhedrin promoter. pFastBacDual-Furin already harbored human Furin, cloned from pGEMfur^68^ (a kind gift from Gary Thomas), under the control of p10 promoter. Coexpression of Furin has previously been shown to be necessary to ensure efficient cleavage of E-Cadherin propeptide in insect cells^69^. Transposition, transfection, virus amplification, protein expression and purification were done the similarly as for EpEX-Fc.

EpFL fusions to fluorescent proteins were cloned into pcDNA3.1 *myc*-His B (Life Technologies). sfGFP^70^ (residues 1–238) and mCherry (residues 1–236) sequences were obtained from iGEM’s Registry of Standard Biological Parts (BBa_K1365020 and BBa_K773003, respectively). N-terminal fusions were prepared in the following manner: EpCAM’s native signal sequence (residues 1–23) with addition of two amino acid residues (QE) to ensure proper cleavage of signal peptide was fused to either sfGFP or mCherry, which was separated from a C-terminally located EpFL (residues 24–314) by amino acid residues GS. C-terminal fusions were comprised of EpFL with its signal peptide (residues 1–314), GS-linker and either sfGFP or mCherry. To generate GFP-Cherry_EX_, sfGFP (1–227) was first fused to mCherry3 via the linker LESGGEDPMVSKGEE. This fusion was then cloned as other N-terminal fusions described above.

### Small angle X-ray scattering (SAXS)

SAXS profiles of ngEpEX were collected at concentrations ranging from 0.5 to 26.2 mg/ml at 15 °C at Stanford Synchrotron Radiation Lightsource (SSRL) Beamline BL4-2 (SLAC National Accelerator Laboratory, Menlo Park, CA). Proteins were purified as described and reference buffer matching was achieved by triple dialysis steps, each overnight at 4 °C against a buffer composed of 20 mM HEPES pH 8.0, 100 mM NaCl, 5% (v/v) glycerol. The 5% glycerol was used for radiation protection of the proteins^71^. All the suspensions were filtered through membranes with 0.1 μm pore size (Millipore, Bedford, MA).

The beam energy and current were 11 keV and 500 mA, respectively. A silver behenate sample was used to calibrate the q-range and detector distance. Data collection was controlled with Blu-Ice^72^. We used an automatic sample delivery system equipped with a 1.5 mm-diameter thin-wall quartz capillary within which a sample aliquot oscillated in the X-ray beam to minimize radiation damage^73^. The sample was placed at 1.7 meter from a MX225-HE (Rayonix, USA) CCD detector with a binned pixel size of 293 μm by 293 μm. Up to twenty 1-second exposures were made for each protein sample and buffer maintained at 15 °C. Each of the diffraction images was scaled using the transmitted beam intensity, azimuthally integrated by SasTool (http://ssrl.slac.stanford.edu/~saxs/analysis/sastool.htm, formerly called MarParse), and averaged to obtain fully processed data in the form of intensity versus q [q = 4πsin(θ)/λ, θ = one-half of the scattering angle; λ = X-ray wavelength]. The buffer profile was subtracted from a protein profile using SasTool. The buffer subtracted SAXS profiles were initially analysed using the ATSAS package^74^ to calculate radius of gyration (*R*_*g*_) and maximum particle size (*D*_*max*_; Supplementary Table 1). Subsequently, the molecular weight of ngEpEX was estimated at multiple concentrations using SAXS MOW^75^. The mean of the smaller scattering angle regions (*q < *0.15 Å^−1^) of the lower concentration profiles (0.5–1.5 mg/ml) and the mean of the wider scattering angle regions (*q* > 0.12 Å^−1^) of the higher concentration profiles (2.0 to 26.2 mg/ml) were merged to obtain the final experimental SAXS profiles. The *ab initio* shape reconstruction for each sample (Fig. 2C) was generated from the corresponding SAXS profile by running DAMMIF^76^ 20 times and then refined through an additional 40 DAMMIN^77^ runs followed by superposition and averaging with DAMAVER^78^.

Molecular envelope was generated using merged SAXS profiles by running DAMMIF^76^ 15 times followed by superposition and averaging with DAMAVER^78^. As EpEX dimer is symmetric, P2 symmetry was assumed however the anisomery level was set to unknown. Final representation of molecular envelope and fitting of EpEX dimer structure was done using UCSF Chimera^79,80^.

### Oligomeric state modeling based on SAXS

Oligomeric state analysis was performed *via* comparison of calculated SAXS profiles to experimental SAXS profile using FoXS^33,34^ and Multi-FoXS^34^, plus by fitting of structure models into the *ab initio* shapes calculated using DAMMIF^76^. Our initial analysis (Fig. 2A,B) showed that calculated *R*_*g*_ and MW values very well correspond to a dimer even at the lowest concentration.

First, a complete model of EpEX dimer was constructed using the EpEX crystal structure (PDB: 4MZV; residues 24–258 were used). C-termini of EpEX dimer corresponding to residue stretch SMQGLK (residues 259–265) along with LE from translated restriction site and His_6_-tag were modeled with MODELER^81^. 30,000 initial models were generated and only top-scoring 10,000 models (according to SOAP score^82^) were used in further analysis. SAXS profiles calculated from dimer structures using FoXS were fitted to merged experimental data in order to get the best fitting model to be used as a building block for tetramer modeling.

Next, tetramer models were generated by combining two dimer structures using PatchDock^36,37^. High accuracy sampling with final clustering set to RMSD 2 Å yielded 44,098 tetramer models with random dimer-dimer orientations. Again, FoXS was employed to calculate their SAXS profiles.

Finally, multi-state modeling with a combined dataset of SAXS profiles calculated from 10,000 dimer and 44,098 tetramer models was done using Multi-FoXS in order to see which combination of the models best describe the experimentally determined SAXS profile. To ensure all possible combinations are considered, χ value percentage threshold for profile similarity was set to 0.1, minimal weight threshold for a profile to contribute to the ensemble was set to 0.01, and the number of combinations expanded to the higher state was set to 100,000. Although the maximal number of states was set to four, fitting stopped at two states, as inclusion of an additional state would fail to significantly improve the fit.

### Chemical cross-linking coupled with mass spectrometry

Purified ngEpEX and ngEpFL protein (equivalent to 700 pmol) was mixed with 5 ×, 25 ×, or 125 × molar excess of disuccinimidyl suberate (DSS) cross-linker (ThermoFisher Scientific) in a final volume of 23 μl of 20 mM HEPES pH 8.0, 100 mM NaCl. The reaction was carried out at 37 °C at 1000 RPM on a thermomixer and stopped after 30 min by adding 1 μl of 1 M Tris pH 8.0 and 8uL of 4 × SDS-PAGE reducing loading buffer (250 mM Tris pH 6.8, 8% SDS, 300 mM DTT, 30% glycerol, 0.02% bromophenol blue). Cross-linked proteins were separated by SDS-PAGE on a 4–20% TGX gel (Criterion) and stained with AcquaStain Protein Stain (Bulldog Bio). Gel bands were excised and diced into 1 mm × 1 mm cubes. Gel pieces were dehydrated three times by adding enough extraction buffer (25 mM NH_4_HCO_3_, 50% ACN) to cover pieces, vortexing for 10 min, and discarding supernatant. Residual liquid was completely dehydrated by SpeedVac. Samples were then reduced and alkylated by first rehydrating gel pieces in enough 15 mM TCEP, 25 mM NH_4_HCO_3_ to cover pieces. Reduction reaction proceeded at room temperature for 20 min and then fresh 0.5 M Iodoacetamide, 25 mM NH_4_HCO_3_ was added to a final concentration of 50 mM. Reaction proceeded in the dark at room temperature for 20 min. Supernatant was removed and gel pieces were washed once with 25 mM NH_4_HCO_3_, then dehydrated with extraction buffer 2 times (vortexting for 5 min). Residual liquid was removed by SpeedVac. Gel pieces were rehydrated in trypsin buffer (0.5 ng/μl mass spec grad typsin in 25 mM NH_4_HCO_3_) with enough buffer to cover pieces (typically 10–50 ng typsin per gel slice). Digest reaction proceeded at 37 °C overnight. Supernatant with resulting peptides was collected, and residual peptides in gel bands were extracted 2 times with 50% ACN, 5% Formic Acid (vortexing for 10 min). A final extraction with 20 μl 100% ACN (vortex 5 min) was collected and all extractions combined to a single extraction tube. Peptides were dried by SpeedVac and kept frozen until LC-MS/MS analysis.

Digested peptides were analyzed by LC-MS/MS using a Thermo Fisher Scientific Easy-nLC 1000 coupled to a dual-pressure linear ion trap (Velos Pro) Orbitrap Elite mass spectrometer (Thermo Fisher Scientific, San Jose, CA). LC-MS/MS was carried out as follows. Prior to LC-MS/MS analysis, peptides were dissolved in 12 μl 3% ACN, 2% Formic Acid. For each sample, 2 μl were loaded onto an online 75 µm × 30 cm fused silica IntegraFrit capillary packed with 1.9 µm Reprosil-Pur C18 AQ reversed-phase resin (Dr. Maisch-GmbH). Peptides eluted by the following gradient program: 5% to 35% ACN, 0.1% formic acid in 10 min, 35% to 95% ACN, 0.1% formic acid in 5 min, and 95% ACN, 0.1% formic acid for 10 min (delivered at a flowrate of 300 nl/min). For each MS cycle, one full MS scan (150–1500 m/z, resolution of 120,000) in the Orbitrap was followed by 20 data-dependent MS/MS scans targeting the twenty most intense ions (with charge state exclusion of all 1+, 2+, and 3+ ions). Selected ions were fragmented by normalized collision energy (setting of 35%) and acquired in the linear ion trap, with any previously acquired ion being dynamically excluded for 20 seconds. Monoisotopic masses of parent ions and corresponding fragment ions, parent ion charge states and ion intensities from LC–MS/MS spectra were extracted using in-house software based on Raw_Extract script from Xcalibur v2.4. Data was searched against a database with the sequences of the EpCAM truncated His-tagged variants, and cross-linked peptides were identified using the Cross-linked Peptide Analysis feature of the BatchTagWeb application on the Protein Prospector search engine (v5.14.2)^39^ DSS was selected as the cross-linker, 20 ppm and 0.5 Da were set as the respective parent and fragment mass tolerances. Default parameters were selected according to the tutorial provided by Protein Prospector (https://vimeo.com/97952461).

### Oligomeric state modeling based on XL-MS

SASD were calculated using Jwalk (version 1.0) algorithm^40^. Two minor modifications were included in the process. First, cross-links with Euclidean distances longer than the cutoff (33 Å) were excluded from SASD calculation to reduce the overall time needed for the analysis. Second, algorithm was modified to account for all possible SASDs of each cross-link, i.e. intra- and inter-subunit.

To each calculated SASD MNXL score was assigned according to published parameters^40^:

$$MNXL\,score\,[SASD]=\{\begin{array}{c}N(18.62,\,35.94)\,\\ -0,1\end{array}\begin{array}{c}if\,SASD\le 33\\ else\end{array}$$

To avoid overscoring of the symmetric models only the best MNXL score was considered, even if there were multiple SASDs calculated for a given cross-link. When scoring tetramer models, the negative penalty for SASD > 33 Å was omitted if the same cross-link had a positive MNXL score in a dimer. The reason for this was to favour the tetramer models matching those cross-links which were non-accessible in the dimer models.

Using this procedure, we scored SASDs using the following models: a monomer model (generated as a subunit of a dimer structure D-21988, described previously), a dimer structure (D-21988), all 44098 tetramer models, 12 D2 symmetric *trans*-tetramer models (generated with Symmdock^37,83^) and 1000 models extracted from corresponding 1000 frames in 10 ns (one frame per 10 ps) MD simulation of monomer a model to account for its flexibility. For simulations NAMD 2.11^84^ with CHARMM-27 force-field was used. Briefly, the monomer model with C-terminus and His_6_-tag modelled in an extended conformation was solvated using a water cube (20 Å margin on each side) and charge-neutralized (addition of Na^+^/Cl^−^ ions). Following minimization for 1000 steps the system was equilibrated for 10 ns (2 fs/step) at 310 K using constant temperature Langevin dynamics, constant pressure via Noose-Hoover Langevin piston and Particle Mesh Edward for full-system periodic electrostatics. Model structures were extracted from the trajectory using VMD^85^.

Additionally, we generated an average occupancy grid for a tetramer model based on Euclidean distances using DISVIS^41,42^. As DISVIS doesn’t address the problem of chain ambiguity which is present in homo-oligomeric cross-links, chain IDs to cross-links non-accessible in the dimer were assigned in a way that the resulting cross-links had the shortest possible distance in the predicted *trans*-tetramer model. Furthermore, symmetry was imposed by adding symmetric cross-links, e.g. for each chain A to chain D cross-link we also added a chain C to chain B cross-link. Average occupancy grid of models which satisfied the highest number of restrains was generated with UCSF Chimera^79,80^.

### Bead aggregation assays

Bead aggregation assays were performed using Fc-chimeric fusion proteins immobilized by the native strong interaction to Protein A-covered magnetic beads. These beads were prepared by coupling Protein A (Sigma Aldrich) to magnetic beads with a diameter of 1 μm (Dynabeads® MyOne™ Carboxylic Acid, Thermo Fisher Scientific) according to manufacturer’s instructions.

Fc-chimeric proteins were incubated together with Protein A-beads for 20 minutes at room temperature, washed twice with PBS buffer containing 0,1% Tween-20, and resuspended in an appropriate buffer. For resuspension of ngEpEX-Fc, gEpEX-Fc and E-CadEX-Fc(Ca^2+^) 20 mM HEPES, pH 8.0, 100 mM NaCl, 2 mM CaCl_2_, 0,1% (v/v) Tween-20 was used. In the case of gEpFL-Fc Tween-20 was replaced by Triton X-100. E-CadEX-Fc(EDTA), was resuspended in the same buffer containing 2 mM EDTA instead of CaCl_2_. Similarly, for evaluating the possible influence of pH on gEpEX-Fc-induced aggregation, a buffer with the same composition without either CaCl_2_ or EDTA was used and the pH value accordingly adjusted; for pH 8,5, HEPES was replaced with Tris-HCl.

Following resuspension beads were incubated with rotation for 30 minutes at room temperature and then transferred to a glass-bottomed slide (µ-Slide 8 Well Glass Bottom, Ibidi) where they were allowed to settle for 30 minutes. Differential interference contrast (DIC) images were captured using Confocal laser scanning microscope Leica TCS SP8 at 600 × magnification. For each sample, five images were used in analysis. Experiments were done in triplicates, starting with the preparation of Protein A-coupled magnetic beads, resulting in total of 15 images per sample.

Image analysis was done using ImageJ^86^. To extract particle sizes the following set of commands was applied: Smooth, Find Edges, set AutoThreshold, Convert to Mask, Fill Holes, and Analyze particles. In each image, 10 single bead particles were manually selected and their average area and standard deviation was calculated. Particles with an area larger than the average plus twice the standard deviation were considered as aggregates^45^. Furthermore, particles with an area smaller than the average minus twice the standard deviation were considered as artefacts and were excluded from the analysis. Bead aggregation ratios were calculated as a ratio between the total area of aggregates divided by the total area of single beads^45^.

### Transfection and treatment of cells for FLIM-FRET

FLIM-FRET experiments were performed using transfected human embryonic kidney cell line HEK 293 T (Thermo Fisher Scientific) and human colon carcinoma cell line HCT8 #L13 (ref.^20^; a kind gift by Olivier Gires) which both lack endogenous EpCAM expression. Cells were cultured in DMEM supplemented with 10% FBS and 1% Pen/Strep. Transfections were preformed using Turbofect (Thermo Fisher Scientific) according to manufacturer’s instructions. In case of GFP_EX_/Cherry_EX_ and GFP_EX_/Cherry_IN_ equimolar amounts of each plasmid was used for transfection. 24 hours after transfection, cells were trypsinized and seeded into wells of µ-Slide 8-Well ibiTreat slides (Ibidi). For FLIM-FRET analysis equal amounts of transfected cells expressing either sfGFP-EpFL or mCherry-EPFL (GFP_EX_, Cherry_EX_ mixed) were thoroughly mixed and seeded into the same well. Cells were then left to grow for 24 hours to reach confluency before FLIM-FRET analysis to ensure formation of cell-cell contacts.

### FLIM-FRET analysis

Cells were fixed with paraformaldehyde solution for 10 minutes. FLIM images of cells were accumulated for 120 s using the SymPhoTime software, which uses TCSPC (time-correlated single-photon counting) technique at 20 MHz. The donor fluorescent protein sfGFP was excited a 488 nm (laser power 2–5%) and fluorescence was detected by HyD SMD detector in range between 500–550 nm. Regions of interest were manually selected to exclude the contribution of internalized proteins. In all cases, except GFP_EX_, Cherry_EX_ mixed, whole cell membrane region was selected. In case of GFP_EX_, Cherry_EX_ mixed, we specifically selected the areas of cell-cell contact, where sfGFP and mCherry signals were colocalized. We achieved the best fitting results by assuming a biexponential decay. In combinations where we observed a significant decrease of average fluorescence lifetime, a triexponential decay was also tested, but the results were not statistically different. Donor lifetime in each combination was determined as amplitude weighted average lifetimes, calculated from the decay fit.

Experiments with all combinations were repeated at least two times, resulting in over 10 cells analyzed in each combination per cell line.

For time-resolved luminescence imaging, the Leica SP8 SMD (Leica Mycrosystems, Manheim, Germany) confocal fluorescence microscope with TCSPC module PicoHarp 300 time-resolved unit (PicoQuant, Berlin, Germany) was used consisting of an inverted microscope (Leica Mycrosystems) equipped with a HCX Plane-Apochromat 63×/ water-immersion CORR CS2 objective with NA 1.2 (Leica, Wetzlar, Germany). A pulsed WLL laser operating at 488 nm was used as an excitation source. The repetition rate was adjusted to 20 kHz. The fluorescence emission (500–550 nm) was guided through a 1 Arry pinhole and was detected with a HyD SMD. Time-resolved luminescence recordings were performed in the time-correlated single-photon counting (TCSPC) mode using a PicoHarp 300 (PicoQuant, Berlin, Germany). In all experiments, the laser power was adjusted to achieve average photon counting rates ≤10^5^ photons/s and peak rates close to 10^6^ photons/s when recording images, thus significantly below the maximum counting rates allowed by TCSPC electronics in order to avoid pile up effects. Data acquisition and analysis were performed by the SymPhoTime64 software (PicoQuant, Berlin, Germany). Thereby, all photons collected in the full frame image were used to form a global histogram for luminescence decay fitting. For deconvolution fitting, the instrument response function (IRF) was measured daily by recording the backscattered excitation light of quenched erythrosine B.

The images were processed with LAS AF software (Leica Microsystems), SymPhoTime SPT software (PicoQuant) and ImageJ^86^. Microscopy images were despeckled, background was subtracted and intensity was adjusted for better visibility. FLIM images were just despeckled and no other manipulations were performed.

### Statistics

Results of BAA and FLIM-FRET experiments are represented as a mean value ± s.d. One-way ANOVA (P < 0.001) with Bonfenorri multiple comparison test was conducted to identify statistically significant differences in mean values.

## Electronic supplementary material

Supplementary Information

## Data Availability

The data that support the findings of this study are available from the corresponding author upon reasonable request.
