# Supplementary material for: EpCAM homo-oligomerization is not the basis for its role in cell-cell adhesion
Source: Sci Rep. 2018 Sep 5;8:13269. doi: 10.1038/s41598-018-31482-7 (PMC6125409; doi:10.1038/s41598-018-31482-7)
Supplement: Supplementary file 1 — Supplementary Information [file 41598_2018_31482_MOESM1_ESM.pdf]

Supplementary Information

EpCAM homo-oligomerization is not the basis for its role in cell-cell adhesion

Aljaž Gaber, Seung Joong Kim, Robyn M. Kaake, Mojca Benčina, Nevan Krogan, Andrej Šali, Miha Pavšič, Brigita Lenarčič

Correspondence should be addressed to M.P. (miha.pavsic@fkkt.uni-lj.si) and B.L. (Brigita.lenarcic@fkkt.uni-lj.si).

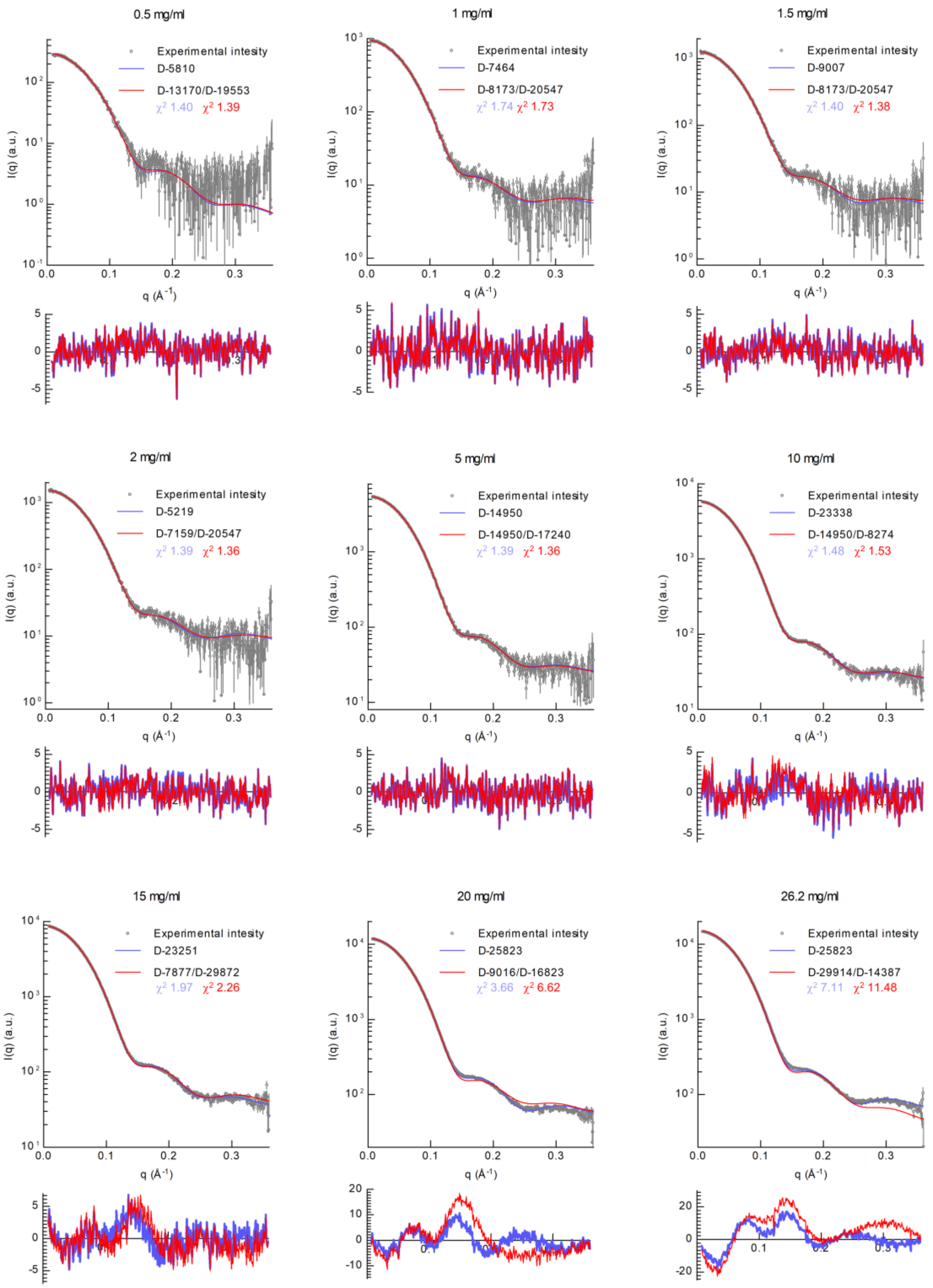

Supplementary Figure 1 | Multi-state modelling with MultiFoXS. Plots represent best single- and two-state model fits, their residuals, and χ^2 values calculated from an ensemble of 10,000 dimer and 44,098 tetramer structure models.

a

Cross-link	Structure/model: monomer	monomer_MD	dimer	PD-14491
	Solvent Accesible Surface Distances (Å)			
33-229	12.76	18.55	13.14	
61-168	24.46	19.90	21.98	
61-218	37.09	33.93		
65-77	38.90	24.48		
77-129	22.15	21.44	21.84	
77-149	44.63	32.56	**22.65 (D)**	
77-151	43.72	33.90	**21.14 (D)**	
77-168	60.63	60.63	44.43	25.53
77-218	25.78	19.28	27.79 (D)	
83-129	19.29	18.52	19.29	
129-129			**19.78 (D)**	
129-151	43.54	37.56	42.72	18.67
151-221	19.94	18.60	18.60	
151-229	26.20	21.49	**20.18 (D)**	
155-168	33.34	25.98	33.15	26.54
155-218	48.57	35.92	34.05	26.60
160-168	16.64	18.62	17.60	
168-229	23.53	18.96	22.07	
**Total MNXL score**	**-0.457**	**/**	**0.277**	**0.154**

Legend

Scoring: Matched; Non-accessible

Color

SASD (Å): 0; 18.62 ± s.d.; 33

b

Model	Total MNXL score	Cross-links, non-accessible in dimer: 155-218	155-168	77-168	129-151
PD-14491	0.154	26.61	26.84	25.52	18.69
PD-43170	0.150	25.13	20.85	28.24	25.76
PD-1442	0.147	25.06	24.97	17.22	31.41
PD-6229	0.137	22.50	29.88	16.76	30.77
PD-329	0.134	25.63	25.06	28.47	24.46
PD-9146	0.134	26.11	22.06	30.81	25.49
PD-1790	0.133	28.61	27.82	25.21	21.43
PD-3308	0.133	27.13	22.36	29.30	24.68
PD-16719	0.127	32.40	20.60	22.70	31.39
PD-14640	0.126	26.12	23.78	32.54	23.85
PD-188	0.126	27.70	25.35	27.39	24.52
PD-9321	0.125	26.65	23.76	30.46	24.49
PD-13802	0.124	27.13	19.74	31.39	27.57
PD-19586	0.123	27.21	19.23	30.07	28.47
PD-2131	0.122	28.13	23.21	28.31	26.04
PD-2922	0.121	28.10	23.30	31.51	24.36
PD-475	0.120	29.15	19.93	30.64	26.75
PD-7435	0.119	30.48	30.47	25.13	20.53
PD-239	0.117	28.54	24.66	28.80	24.31
PD-31436	0.116	30.40	22.69	24.10	30.29
PD-600	0.116	25.95	24.93	30.45	25.17
PD-21236	0.113	27.20	21.20	32.23	27.42
PD-3343	0.113	25.35	30.04	27.14	25.01
PD-25406	0.111	27.22	22.29	32.08	27.72
PD-19970	0.108	31.24	17.91	27.76	29.18
D2-16	-0.130	50.49	24.19	32.85	/
D2-11	-0.208	45.68	17.96	44.96	/
D2-19	-0.217	44.48	21.48	45.09	/
D2-14	-0.262	48.12	27.75	37.34	/
D2-12	-0.265	46.00	29.71	37.84	/
D2-18	-0.276	38.35	32.02	/	/
D2-22	-0.280	31.38	41.78	/	/
D2-15	-0.387	38.00	/	/	/
D2-17	-0.391	39.11	34.39	/	/
D2-24	-0.396	35.55	40.03	/	/
D2-23	-0.398	35.50	/	/	/
D2-25	-0.400	37.96	37.80	/	/

Supplementary Figure 2 | Cα-Cα Solvent Accessible Surface Distances. (**a**) Comparison of distances in different monomeric, dimeric and oligomeric (PD-14491) models. Best distances during 10 ns MD simulation of monomer flexibility (monomer_MD) are also presented for comparison. In dimer structure, inter-molecular distances are bolded and assignated with (D). (**b**) Comparison of MNXL score and SASD between random and D2 symmetric tetramer models. Table contains scores and distances 25 best scoring random tetramer models and all generated D2 symmetric *trans*-tetramer models.

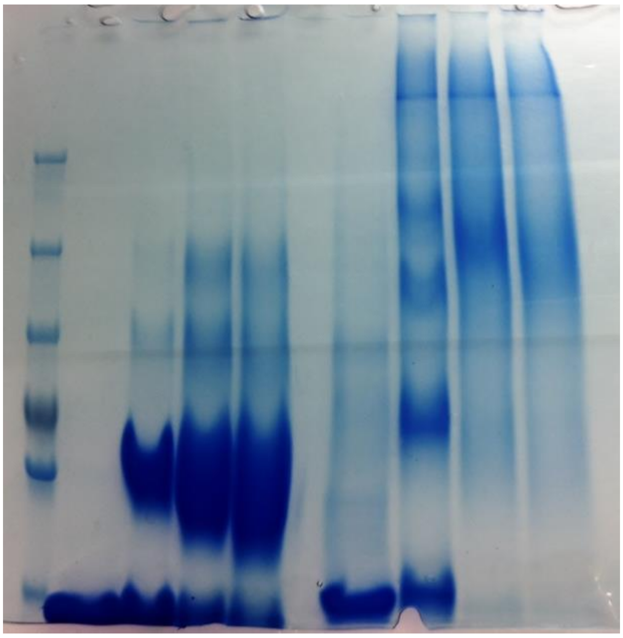

Supplementary Figure 3 | SDS-PAGE analysis of cross-linking experiment. Full-length gel of results presented in Figure 3a. PageRuler Plus Prestained Ladder (Thermo Fisher Scientific) was used as molecular size marker.

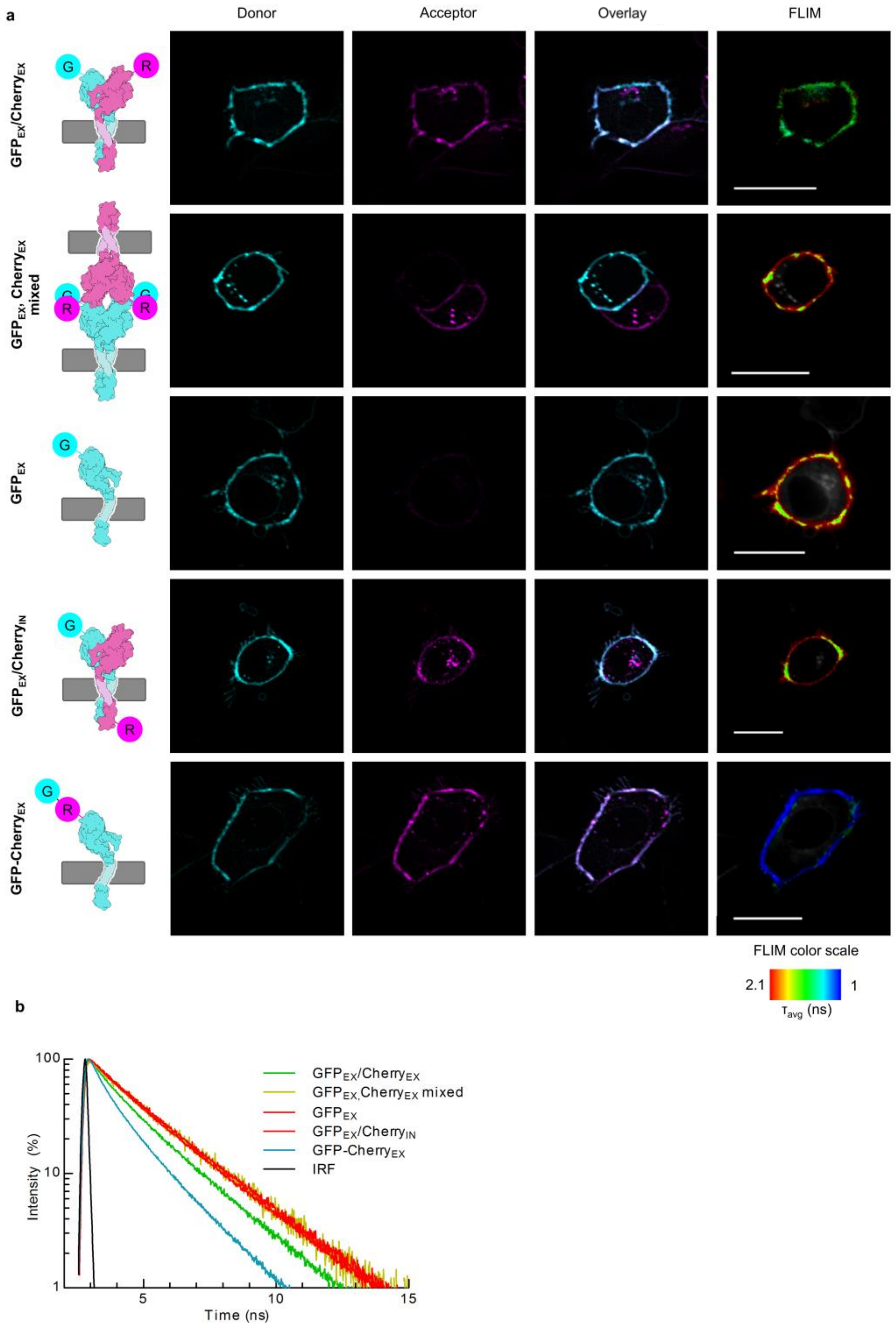

Supplementary Figure 4 | FLIM-FRET (HCT8^{EpCAM-}). (**a**) Analysed combinations of fluorescently tagged EpCAM proteins. sfGFP (G) fluorescence is colored cyan and mCherry (R) fluorescence is colored magenta. The same color scheme applies to schematic representations on the left. White line represents 20 μm. FLIM color scale is the same in all presented FLIM measurements. (**b**) Representative fluorescence lifetime decays for each analysed combination.

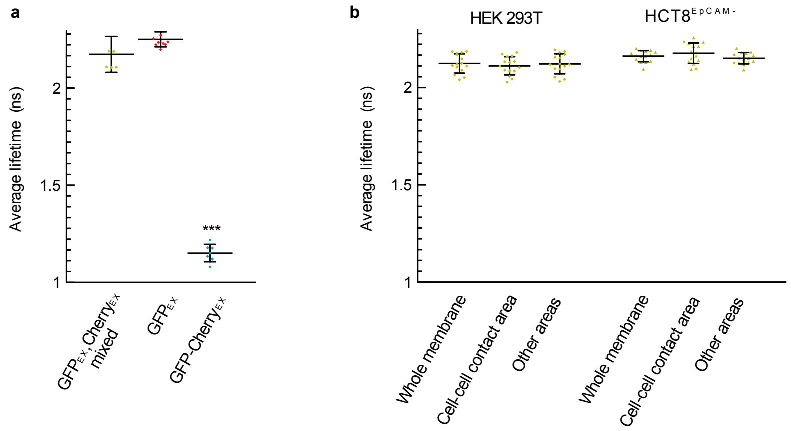

Supplementary Figure 5 | FLIM-FRET in live cells and FLIM-FRET as a function of position in the membrane in GFP$_{EX}$, Cherry$_{EX}$ mixed. (**a**) Mean lifetimes with s.d. of measurements performed in live HEK 293T cells, ***$p < 0.001$, one-way ANOVA test with Bonferroni post hoc analysis, compared to mean values of negative control (GFP$_{EX}$). (**b**) Mean lifetimes with s.d. of different regions of cell membranes in combination GFP$_{EX}$, Cherry$_{EX}$ mixed in fixed HEK 293T and HCT8^{EpCAM-} cells. Other areas are all areas of cell membrane other than cell-cell contact area.

a

c (mg/ml)	**0.5**	**1**	**1.5**	**2**	**5**	**10**	**15**	**20**	**26.2**	**Dimer**
$MW_{SAXS MOW}$ (kDa)	55.1	57.8	58.1	57.8	57.9	58	57.1	55.7	54.6	**57**
R_g (Å)	24.9	25.2	25.2	24.9	24.7	24.8	24.5	24.1	23.8	**24**
c^2	1.40	1.74	1.40	1.39	1.39	1.48	1.97	3.66	7.11	/

b

c (mg/ml)	**0.5**	**1**	**1.5**	**2**	**5**	**10**	**15**	**22.8**	**Dimer**
$MW_{SAXS MOW}$ (kDa)	55.8	56.1	56.7	57.3	59.2	59.0	58.8	56.7	**57**
R_g (Å)	24.9	25.2	25.5	25.2	25.1	25.2	25.2	24.6	**24**

c

c (mg/ml)	**0.5**	**1**	**1.5**	**2**	**5**	**10**	**16.9**	**Dimer**
$MW_{SAXS MOW}$ (kDa)	52.8	56	55.9	56.9	58.2	58.6	57.8	**57**
R_g (Å)	25.8	25.3	25.2	25.3	25.2	25.0	24.8	**24**

Supplementary Table 1 | SAXS analysis. (**a**) Comparison of MW and R_g values calculated from experimental SAXS profiles with those from the dimer X-ray structure (PDB: 4MZV). Also, the χ^2 fit values of the dimer structure to the SAXS profiles are shown. (**b**) and (**c**) show MW and R_g values calculated from experimental SAXS profiles recorded in buffer without glycerol, and buffer containing 1 % (w/v) sucrose and 5 mM Na/K Nitrate, respectively.
